# Thyrotoxicosis Periodic Paralysis: A Rare Presentation of a Common Disease

**DOI:** 10.7759/cureus.25551

**Published:** 2022-05-31

**Authors:** Jawahar Al Noumani, Zubaida S Al Falahi, Hatem Farhan, Abdullah M Al Alawi

**Affiliations:** 1 Internal Medicine, Oman Medical Specialty Board (OMSB), Muscat, OMN; 2 Medicine, Sultan Qaboos University Hospital, Muscat, OMN

**Keywords:** hypophosphatemia, hypokalemia, graves’ disease, thyrotoxicosis, periodic paralysis

## Abstract

We report a 31-year-old man of an Arabic ethnicity who presented to the Emergency Department (ED) with a one-night history of progressive generalized weakness followed by an inability to move all four limbs. The patient was found to have hypokalemia and hypophosphatemia. Detailed inpatient assessment revealed that the patient had undiagnosed Graves' disease with thyrotoxicosis causing electrolyte disturbances and paralysis. The patient's symptoms resolved after the correction of the electrolytes. In this case study, we report an unusual presenting symptom of paralysis of Graves' disease in a patient of Arabic ethnicity.

## Introduction

Periodic paralysis is a rare group of diseases causing muscle weakness due to muscle ion channelopathy [1]. It is triggered by high carbohydrate meals, heavy exercise, or fasting. It can be subclassified as hyperkalemic or hypokalemic. Both hereditary and acquired causes have been identified. The main hereditary causes are hypokalemic periodic paralysis, hyperkalemic periodic paralysis, and Andersen syndrome.

Inherited periodic paralysis disorders are caused by autosomal dominant defects in calcium, sodium, or potassium channels [2,3]. Thyrotoxicosis periodic paralysis is the only reported acquired cause of periodic paralysis. It occurs more in Asian males in their 30s and mostly during summertime [4-6]. Around 2% of patients with hyperthyroidism develop periodic paralysis, and Graves' disease is the leading cause in most cases [7-9]. There were only two cases of thyrotoxicosis periodic paralysis reported from Arabic ethnicity [10,11]. We are reporting the third case of periodic paralysis as a first manifestation of thyrotoxic Graves' disease in a patient of Arabic ethnicity. This case study highlights the manifestation of periodic paralysis in patients with thyrotoxicosis and the course of management for effective recovery.

## Case presentation

A 31-year-old man with no previous medical background presented to the emergency department (ED) on May 31, 2021, with a history of tingling and cramps on both lower limbs that started after carrying a heavy object. The cramps progressed overnight, and the patient could not move his lower limbs when he woke up in the morning of the next day due to generalized body weakness. He was brought to the hospital, and upon arrival at the ED, the patient complained of paralysis associated with chest heaviness and difficult breathing. There was no proceeding febrile, diarrheal, or respiratory illness. He did not complain of headache, dizziness, loss of consciousness, abnormal movement, neck stiffness or pain, visual or hearing disturbance, tongue heaviness, or facial asymmetry. He denied any trauma or fall. He was conscious, alert, and oriented to time, place, and person, with preserved speech. His vitals were as follows: temperature, 36.9 centigrade; heart rate, 78 beats per minute; respiratory rate, 18 breaths per minute; blood pressure, 133/77 mm Hg; and oxygen saturation of 99% on ambient air.

The cranial nerves examination was unremarkable. The power was 2/5 in all four limbs, with absent reflexes, but normal muscle tone and plantar reflexes were equivocal. Likewise, the musculoskeletal, cardiovascular, and respiratory examinations were unremarkable. Computed tomography (CT) scan of the brain was normal. Other relevant laboratory investigation results are shown in Table 1. In summary, he has hypokalemia and hypophosphatemia.

**Table 1:** Laboratory investigations on presentation and at the time of discharge from the hospital CRP: C-reactive protein; ALT: Alanine aminotransferase; AST: Aspartate aminotransferase; ALP: Alkaline phosphatase; TSH: Thyroid-stimulating hormone; eGFR: Estimated glomerular filtration rate.

Test	Result on admission	Result on discharge	Reference range
Hemoglobin	14.7	-	11.5-15.5 g/dL
White cell count	6.4	-	2.2-10.0^9^/L
Neutrophils	5.6	-	1.0-5.0 10^9^/L
Platelets	319	-	150-450 10^9^/L
C-reactive protein	12	-	0-5 mg/L
Sodium	139	139	135-145 mmol/L
Potassium	2.4	4.5	3.5-5.1 mmol/L
Creatinine	90	83	59-104 μmol/L
eGFR	85	>90	>90 mL/min/1.73 m^2^
Calcium	2.50	-	2.15-2.55 mmol/L
Phosphate	0.37	1.73	0.81-1.45 mmol/L
Magnesium	0.79	0.94	0.66-1.07 mmol/L
ALP	89	-	35-104 U/L
Bilirubin	19	-	0-17 mg/dL
TSH	<0.01	0.01	0.27-4.2 mIU/L
Free T4	57.2	21.0	13.1-21.3 pmol/L
Random morning cortisol	54	293	nmol/L
Short synacthen test (3 days after admission)	0 min = 293, 30 min = 438, 60 min = 498	-	nmol/L
Creatine kinase (CK)	64	-	39-308 U/L
Urine potassium (random)	41	-	20-40 mmol/L
Thyroid antibodies	44	-	0-50 IU/ml
ALT	30	-	0-33 U/L
AST	14	-	0-32 U/L

**Figure 1:**
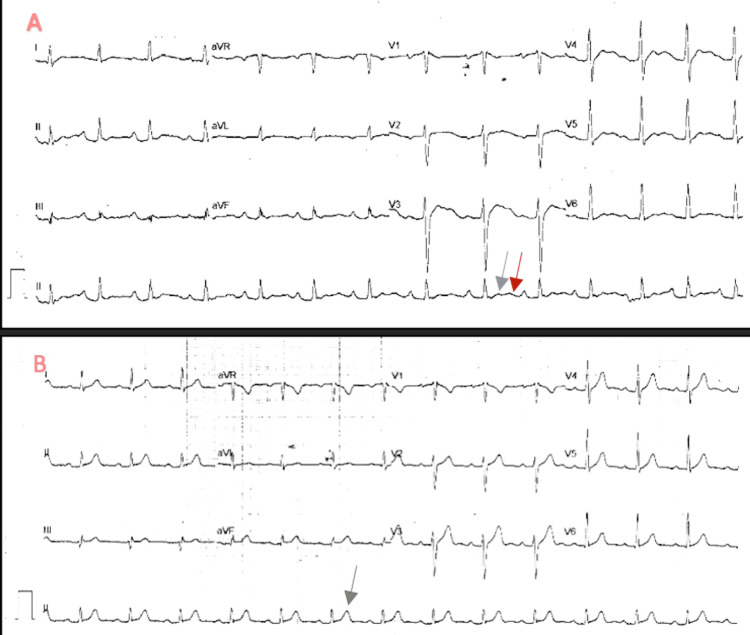
(A) An initial ECG on arrival: regular sinus rhythm with a heart rate of 100 beats/ minute as well as flattened T wave (gray arrow) and U wave (red arrow). (B) ECG after electrolytes correction showing the normalization of T wave (arrow) and disappearance of U wave. ECG: Electrocardiogram.

Also, an electrocardiogram (ECG) (Figure 1) showed regular sinus rhythm with HR 100 and flattened T wave, U wave, QTc 343, and no ST depression or premature ventricular contractions (PVCs).

**Figure 2:**
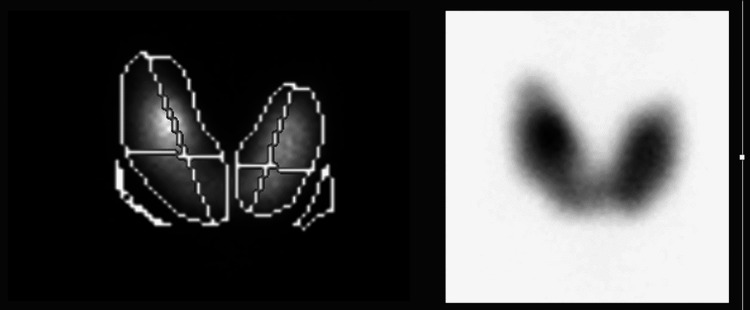
Thyroid scintigraphy with Tc-99m showing typical features of Grave’s disease and a diffusely enlarged thyroid gland with homogenously increased uptake and suppression of the background

The patient received intravenous (IV) potassium chloride and potassium phosphate corrections. Four hours later, the chest heaviness resolved; he moved both the upper limbs with power 5/5 and moved his lower limbs bilaterally with mild proximal weakness, which resolved later during the first day of admission. As shown in Table 1, the patient had hyperthyroidism, so an ultrasound of the thyroid was done, which showed increased vascularity. Furthermore, thyroid uptake scan NM (nuclear medicine) thyroid "99mTc" (Figure 2) showed an enlarged gland with diffuse homogenous increased radiotracer total uptake of 5.8% (normal 1%-4%). Therefore, the patient was commenced on carbimazole (20 mg twice per day) and propranolol (40 mg twice per day) to treat Graves' disease.

The patient was reviewed in the endocrinology clinic, and he remains well and asymptomatic. Also, he had normalization of the thyroid function test, so the carbimazole dose was reduced to 10 mg daily.

## Discussion

This case demonstrated unusual presentation - hypokalemic periodic paralysis - of a common disease, Graves' disease. Also, it shared the management principles that included immediate correction of electrolyte derangements and management of the underlying disease. Predisposition to thyrotoxicosis periodic paralysis can be due to the presence of ion channel defects that affect *KCNJ2 *gene expression. This gene encodes the Kir2.6 potassium channel in skeletal muscle, which is regulated by thyroid hormone [12-14]. Also, thyrotoxicosis increases tissue responsiveness to insulin and epinephrine, which increases sodium-potassium ATPase activity on the skeletal muscle membrane leading to several electrolyte derangements, including hypokalemia and hypophosphatemia. This effect is more seen in those with Graves' disease. Those patients usually require close monitoring of their electrolytes and ECG [15-17].

Thyrotoxicosis periodic paralysis is usually treated acutely by treating electrolyte disturbance. Symptoms take hours to days to resolve [18]. If hypokalemia and weakness are refractory, intravenous propranolol is an effective second-line treatment [19]. Further, treatment to prevent recurrence is by controlling the cause of hyperthyroidism to achieve a euthyroid state, and propranolol can be used as a preventive measure as well [20]. Our patient presented with acute muscle weakness of four limbs with hypokalemia and hypophosphatemia associated with ECG changes. Further testing showed thyrotoxicosis with Grave's picture in the thyroid uptake scan. All his complaints were fully reversed by electrolytes correction on day one, and his thyrotoxicosis was treated with a long-term plan.

## Conclusions

Thyrotoxicosis should be considered in patients presenting with clinical and biochemical pictures of periodic paralysis, and the initial management should be directed to correct the electrolyte derangement. The associated weakness is fully reversible by electrolytes correction, and further attacks can be prevented by appropriate management of thyrotoxicosis.
